# Identification of Genes Regulating Gene Targeting by a High-Throughput Screening Approach

**DOI:** 10.4061/2011/947212

**Published:** 2011-02-13

**Authors:** Fabien Delacôte, Christophe Perez, Valérie Guyot, Catherine Mikonio, Pierrick Potrel, Jean-Pierre Cabaniols, Christophe Delenda, Frédéric Pâques, Philippe Duchateau

**Affiliations:** ^1^Cellectis SA, 102 Avenue Gaston Roussel, 93340 Romainville Cedex, France; ^2^Cellectis Bioresearch, 102 Avenue Gaston Roussel, 93340 Romainville Cedex, France

## Abstract

Homologous gene targeting (HGT) is a precise but inefficient process for genome engineering. Several methods for increasing its efficiency have been developed, including the use of rare cutting endonucleases. However, there is still room for improvement, as even nuclease-induced HGT may vary in efficiency as a function of the nuclease, target site, and cell type considered. We have developed a high-throughput screening assay for the identification of factors stimulating meganuclease-induced HGT. We used this assay to explore a collection of siRNAs targeting 19,121 human genes. At the end of secondary screening, we had identified 64 genes for which knockdown affected nuclease-induced HGT. Two of the strongest candidates were characterized further. We showed that siRNAs directed against the ATF7IP gene, encoding a protein involved in chromatin remodeling, stimulated HGT by a factor of three to eight, at various loci and in different cell types. This method thus led to the identification of a number of genes, the manipulation of which might increase rates of targeted recombination.

## 1. Introduction

The transfection of cells with exogenous DNA can be used to generate stable transformants with the exogenous sequence integrated into their genomes by random insertion (RI) or targeted integration driven by homologous recombination. In the case of homologous recombination, the integration of DNA into the genome is referred to as “homologous gene targeting” (HGT). Both RI and HGT depend on DNA double-strand break (DSB) repair mechanisms. 

DSBs are particularly hazardous events in cells. Two different and competing mechanisms repair DSBs. Homologous recombination (HR) involves the use of homologous sequences as a template for restoring genomic integrity upon DSB induction and is considered to be an error-free mechanism. Genetic and biochemical studies have shown that HR in yeast is mediated by the *RAD52* epistasis group of genes [1], which are required to various extents for HGT. For example, almost no HGT is observed in the absence of a functional *RAD52* gene, but substantial levels of recombination are observed in absence of *RAD51* and *RAD57* [2]. Homologs of these genes have been identified in vertebrates, including *RAD51, RAD51B, RAD51C, RAD52, RAD54, XRCC2*, and *XRCC3,* which have been shown to be necessary for HGT in the DT40 chicken lymphoid cell line [3–7]. In mouse embryonic stem (ES) cells, HGT is decreased slightly by mutations in *BRCA2* [8], strongly by mutations in *BRCA1* [9] and *RAD54* [10] and is completely abolished by mutations in *ERCC1*/XPF [11]. By contrast, nonhomologous end joining (NHEJ) requires little or no homology for DSB repair and is often associated with insertions and/or deletions at the DSB site [12]. This mechanism is therefore considered to be error prone. Several factors involved in NHEJ have been identified, including the Ku DNA-binding heterodimers, the DNA-PKcs protein kinase, Artemis, DNA ligase IV (Lig4), and associated partners XRCC4 and XLF/Cernunnos [12]. These proteins differ in their impact on the efficiency of the NHEJ process, but the absence of XRCC4, Lig4, and Ku strongly increases the proportion of error-associated DSB repair events [13–16]. Early studies reported that Chinese hamster cell lines with mutations in *XRCC4* (xrs-1) or *Ku80* (xrs-6) had lower random integration frequencies than the wild type (WT) [17, 18]. Furthermore, mouse cells with a mutant DNA ligase IV were found to have significantly lower random integration frequencies than WT cells [19]. Finally, Ku80 and DNA ligase IV deficiencies in plants lead to defective T-DNA integration [20]. All these findings are consistent with a role for NHEJ in random integration events. However, the precise mechanism of random integration remains to be determined.

HGT provides the ultimate genetic tool for investigating gene function, as it can be used for the specific modification of almost any genomic sequence. Moreover, HGT may provide an alternative approach for gene therapy strategies, because targeted integration into a genomic safe harbor may reduce the risks of insertional mutagenesis. However, the low frequency of homologous gene targeting has been a major obstacle to the use of this technology. Indeed, random integration appears to be the major DNA integration pathway in most organisms, including mammals and higher plants (for review, see [21]), with the exception of *S. cerevisiae* and a handful of other species and cell types. Several reports have also indicated that HR is efficient essentially during the late S and G2 phases of the cell cycle [22–29], suggesting that it may be difficult to make use of HGT in postmitotic cells. 

Various approaches have been tested for improving gene targeting performances. Selection procedures have been used, to eliminate unwanted random integration [30, 31]. However, although these methods increase the yield of targeted events in transformants, they have no impact on absolute gene targeting frequencies—the number of targeted events per transfected cell. Many other studies have aimed to increase the efficiency of HR. One of the most successful methods in current practice is based on the use of rare cutting endonucleases, such as meganucleases or zinc finger nucleases (ZFNs) to induce a DSB in the targeted gene [21]. Meganucleases are natural endonucleases that induce targeted recombination in living cells [32, 33], whereas ZFNs are generated by fusing a zinc finger-based DNA-binding protein with the catalytic domain of the bacterial FokI endonuclease [34–36]. Robust stimulation of HGT by a factor of 100, or even 1000, has been achieved by several groups in this way, resulting in several percents of targeted events in immortalized cells [37–41]. However, the degree of stimulation achieved is highly variable and depends on the nuclease [37] and other factors. For example, a zinc finger protein recognizing a sequence present in the plant SurA and SurB genes has been shown to induce different levels of targeted mutagenesis in these two genes [38], suggesting that target accessibility or the efficacy of a DNA repair pathway may be locus-dependent. The efficiency of the process also depends on cell type: by using nonintegrative lentiviral vectors rather than transfection, Lombardo et al. induced up to 50% HGT events at the CCR5 locus in K562 and Jurkat cells, about 5% in ES cells, and almost 0.1% in CD34+ cord blood progenitor cells [37]. Similarly, Zou et al. achieved much lower levels of DSB-induced recombination at the *PIG-A* locus in human ES cells and induced pluripotent stem cells (iPS) (2–4 × 10^−4^ and ×10^−5^, resp.) than in 293 cells (3%) [40]. These differences may be due to lower efficiencies of vectorization and/or nuclease expression, differences in the likelihood of homologous recombination in different cell types, or a combination of these factors. Nevertheless, there is still room for improvement. Given the cell cycle dependence of homologous recombination, Urnov et al. and others have induced simple reversible cell cycle arrest in the G2 phase with reagents such as vinblastine, resulting in a small but significant increase in nuclease-induced HGT in immortalized cell lines [38, 39].

Another approach involves direct modulation of the cellular DNA repair machinery. Several groups have adopted this strategy and have tried to stimulate HGT by overproducing DNA repair proteins [42–46]. Overexpression of the human *RAD51* gene resulted in a two to three times increase in HGT [43], but higher rates (10 to 64 times higher) have been reported in other studies involving overproduction of the bacterial RecA [44] or yeast Rad52 [46] proteins in mammalian cells or of yeast Rad54 in *Arabidopsis* [45]. Other groups have investigated the impact of NHEJ inhibition on HGT, with conflicting results in some cases. Pierce et al. observed that HGT frequencies were similar in wt, *Ku70^−/−^, DNA-PK^−/−^*, and *Xrcc4^−/−^* mouse ES cells [47]. Similarly, Domínguez-Bendala observed a similar absence of HGT stimulation in *Ku80*- and *DNA-PK*-deficient cells, although they did observe a significant increase in HGT following the knockdown of *PARP-1*, an unrelated gene [48]. By contrast, downregulation of the *Ku70* and *XRCC4* genes by siRNAs in human cells leads to significant 30 times increase in the HGT/RI ratio at the *HPRT* locus [49].

By combining the use of a nuclease to create a DSB in the targeted gene and the modulation of specific genes, it should be possible to achieve the synergistic stimulation of HGT. Various studies have shown that the efficiency of homologous recombination induced by a nuclease could further be enhanced by the overproduction or inactivation of DNA repair proteins [47, 50, 51], although all these studies involved the *a priori* identification of potential candidates on the basis of their role in DNA repair. Several genome-wide RNAi screening analyses have recently improved our understanding of the DNA repair pathway [52–54]. Słabicki et al. identified 61 genes affecting DNA DSB repair in human HeLa cells [54]. The downregulation of 17 of these genes led to an increase in endonuclease-induced homologous recombination. However, this study assessed intrachromosomal recombination rather than gene targeting *per se* (recombination between an exogenous DNA sequence and a target chromosomal locus). It therefore seems likely that many other genes may be involved in the DSB-induced HGT mechanism. 

We have developed a high-throughput screening system for a genome-wide study of factors affecting nuclease-induced HGT. Using an assay monitoring the HGT induced by the I-SceI meganuclease, we screened a library of siRNAs and identified several genes for which downregulation was associated with a significant increase in HGT efficiency. Sixty-four candidates were confirmed by secondary screening. Two of these candidates, *EP300* and *ATF7IP*, which are not known to be directly involved in DNA repair, were characterized further. We show here that the downregulation of these genes leads to a significant increase in the frequency of HGT at endogenous loci in human cells.

## 2. Materials and Methods

### 2.1. Cell Culture

The GM00847, cGPS HEK-293, and 293H cell lines were cultured in Dulbecco's modified Eagle's medium (dMEM) plus Glutamax supplemented with 10% fetal calf serum, 2 mM L-glutamine, 100 IU/ml penicillin, 100 *μ*g/ml streptomycin, and 0.25 *μ*g/ml amphotericin B (Fungizone) at 37°, under an atmosphere containing 5% CO_2_. cGPS HEK-293 cells were grown in the presence of 0.1 mg/ml hygromycin B (Sigma). The E2 clone for the measurement of I-SceI-induced gene targeting with the luciferase reporter system was selected and maintained on 400 and 250 *μ*g/ml G418, respectively. Finally, the clone used for measuring I-SceI-induced gene targeting with the GFP reporter system was selected on 400 *μ*g/ml G418 and 0.4 *μ*g/ml puromycin. For cellular expansion, this clone was maintained in medium containing 200 *μ*g/ml G418 and 0.2 *μ*g/ml puromycin.

### 2.2. Generation of Cell Lines for Primary and Secondary Screening

**Figure 1:**
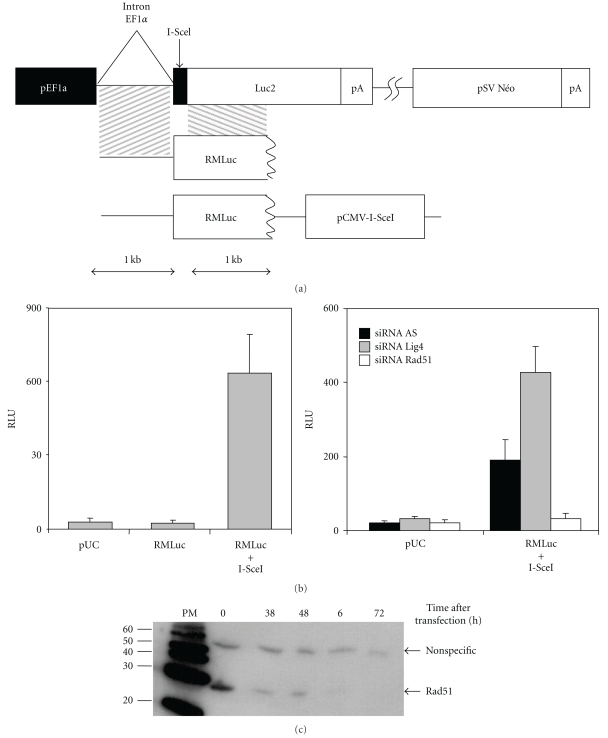
A cell-based assay for identifying factors stimulating I-SceI-induced HGT. (a) A reporter system for I-SceI-induced gene targeting. The firefly luciferase gene (Luc2) is inactive due to replacement of the first 22 base pairs (bp) by a 24 bp I-SceI site (vertical black box). The plasmids constructed for I-SceI-induced gene targeting are described as follows: RMLuc+I-SceI has (i) the first 22 bp of the luciferase gene surrounded by 1 kb of homologous sequence (hatched boxes); (ii) an I-SceI induction cassette under the control of a CMV promotor. The RMLuc plasmid does not contain the I-SceI expression cassette. (b) Validation of the cell-based assay. The E2 cell line carrying a single integrated copy of the reporter system described in (a) was transfected with empty vector (pUC), RMLuc, or RMLuc+I-SceI (left panel). Luciferase activity was analyzed 72 hours after transfection. The E2 cell line was also cotransfected with either pUC or with RMLuc+I-SceI plus siRNAs known to modulate gene targeting (right panel): *RAD51* siRNA and *LIG4* siRNA. The results obtained were compared with those for cotransfection with the control All Star (AS) siRNA. Luciferase activity was assessed 72 hours after transfection. (c) Western blot analysis of Rad51 protein levels at various times after transfection, for the E2 clone with RMLuc+I-SceI and *RAD51* siRNA. Specific and nonspecific bands were detected at 30 and 50 kDa, respectively.

For primary screening, we generated a cell line derived from GM00847 (Coriell Institute) carrying a luciferase-based reporter gene, as described in Figure 1(a). We electroporated 10^6^ GM00847 cells with 500 ng of the gene-targeting substrate plasmid (Figure 1), linearized by *Pvu*I digestion. Electroporation was carried out with the Amaxa kit, according to the manufacturer's instructions. Two days after transfection, 400 *μ*g/ml G418 were added to the cells. The selected clones were then amplified for genomic DNA extraction, for Southern blots to determine whether the transgene was present as a single copy. 

For secondary screening, we generated a cell line derived from cGPSHEK293 (Cellectis Bioresearch, Romainville, France) carrying a GFP-based reporter gene, as described in Figure 3(a). We used 10^6^ cells to seed 10 cm tissue culture dishes on the day before transfection. We cotransfected cells with 3 *μ*g of gene targeting substrate and 2 *μ*g of I-CreI expression plasmid using Lipofectamine 2000 reagent (Invitrogen), according to the manufacturer's instructions. Twenty-four hours after transfection, the culture medium was replaced with fresh medium supplemented with 0.4 mg/ml G418. After 12 days of selection, the second selective agent, puromycin, was added at a concentration of 0.4 *μ*g/ml. After incubation for seven to nine days in the presence of both selective agents, single-colony clones were picked in 96-well plates. Double-resistant clones were analyzed by Southern blotting for integration at the I-CreI locus (data not shown).

### 2.3. Screening Assays

For primary screening, 14,000 cells per well were seeded in white 96-well plates on the day before transfection. Cells were transfected with 200 ng of Luciferase Repair Matrix and I-SceI induction plasmid (RMLuc+I-SceI see Figure 1(b)) per well or with Luciferase Repair Matrix alone (RMLuc, see Figure 1(b)), with or without 33 nM siRNA, in the presence of 0.8 *μ*l of Polyfect transfection reagent (Qiagen). We added 50 *μ*l of ONEGlo (Promega) to each well, 72 hours after transfection, and incubated the cells in the dark for 3 minutes before luciferase activity analysis (1 second/well) with a PHERAStar luminometer (BMG Labtech). For secondary screening, 15,000 and 10^6^ cells were seeded in 96-well plates or 10 cm dishes, respectively, on the day before transfection. Cells were transfected with 200 ng (for 96-well plates) or 5 *μ*g (for 10 cm dishes) of RMGFP+ISceI (Figure 3) with or without siRNA at a final concentration of 33 nM, using 1.35 *μ*l and 90 *μ*l of Polyfect transfection reagent, respectively. Cells were trypsinized 96 hours after transfection, and the percentage of GFP-positive cells was monitored by flow cytometry (Guava Instruments). We monitored siRNA transfection efficiency with rhodamine-labeled siRNA coupled with flow cytometry detection. Transfection efficiency reached 73%, indicating that the transfection conditions used were very good (data not shown). The ratio of the percentage of GFP+ cells for a specific siRNA over that for the siRNA control All Star (AS Qiagen) was used to determine the stimulation factor for each specific siRNA. The potential effect of siRNAs was assessed by applying Student's *t*-tests to the stimulation factor. Two controls siRNAs were used to validate siRNA transfection: the *RAD51* and *GFP* siRNAs. The *RAD51* and *GFP* siRNAs decreased the percentage of EGFP-positive cells by factors of six and four with respect to the control All Star (AS) siRNA, demonstrating that the siRNAs were active (Figure 4). All experiments carried out in 96-well plates (cell seeding, cell transfection, incubation, and luciferase detection) were performed with a Velocity 11 robot (Velocity, Palo Alto, CA). *Z*-scores were calculated with the following equation, *Z* = (*x* − *μ*)*σ* ^−1^, where *x* is the mean luciferase signal for a given siRNA, *μ* is the mean luciferase signal for the run, and *σ* is the standard deviation of the run. Means and standard deviations were calculated excluding controls.

### 2.4. Targeted Integration at the hRAG1 Locus

On the day before transfection, 10^6^ 293H cells were seeded in 10 cm dishes. Cotransfection was performed with 3 *μ*g of meganuclease expression plasmid, 2 *μ*g of Knock-In matrix, and 33 nM siRNA, in the presence of 90 *μ*l of Polyfect. The Knock-In plasmid has all the necessary characteristics favoring highly efficient homologous recombination at the endogenous hRAG1 locus in 293H cells. The left and right arms correspond to isogenic sequences of 2 kb and 1.2 kb in size, respectively, surrounding the RAG1 meganuclease recognition site. These two homologous arms are separated by a heterologous 1.7 kb fragment [55].

Twenty-four hours after transfection, cells were trypsinized and seeded in 96-well plates at a density of 10 cells per well. Two weeks after transfection, genomic DNA was extracted with the ZR96 Quick-gDNA kit (Zymo Research), according to the manufacturer's protocol. We screened for knock-in events by PCR, as described by Grizot et al. [55], using oligonucleotides F2: 5′-AGGATCTCCTGTCATCTCAC-3′ and R12: 5′-CTTTCACAGTCCTGTACATCTTGT-3′. The percentage of knock-in events was calculated, taking into account the plating efficiency for transfected cells, which was estimated at 30% (data not shown).

### 2.5. Targeted Integration at the hXPC4 Locus

On the day before transfection, 10^6^ 293H cells were used to seed 10 cm dishes. Cotransfection was performed with 3 *μ*g of meganuclease-encoding plasmid, 2 *μ*g of Knock-In matrix, and 1 nM siRNA, using 25 *μ*l of Lipofectamine 2000, according to the manufacturer's protocol. The Knock-In plasmid for the XPC 293H endogenous locus has left and right arms corresponding to isogenic sequences of 1.6 kb and 1.5 kb in size surrounding the hXPC4 meganuclease recognition site. These two homologous arms are separated by a 4.7 kb heterologous fragment of DNA containing a functional neomycin resistance gene under the control of a CMV promoter. The cells were trypsinized 72 hours after transfection, and 2000 cells per plate were used to seed 10 cm dishes. One week after seeding, we added 400 *μ*g/ml G418 to the cells. One week later, G418-resistant clones were picked and amplified in 96-well plates. Genomic DNA was extracted with the ZR96 Quick-gDNA kit (Zymo Research), according to the manufacturer's protocol. The percentage of targeted events in stable transformants was analyzed by PCR screening with the oligonucleotides F3: 5′-CAAGCACCATAACAAACAACATTGA-3′ and R1: 5′-ATCCGAAAATGGATATACAAGCTC-3′ (cf.  Figure 7).

### 2.6. Western Blot Analysis

All stages of protein extract preparation were carried out at 4°. Cells were washed with PBS, suspended in RIPA lysis buffer with 2 mM PMSF, 1 mM orthovanadate sodium, and protease inhibitor cocktail (Santa Cruz) and incubated for 30 minutes on ice. Extracts were centrifuged at 15,000 g for 30 minutes. The supernatant was retrieved, and its protein concentration was determined with the BCA protein assay. Boiled protein extract (25 *μ*g) was subjected to SDS-PAGE in a 10% polyacrylamide gel. After migration, the proteins were electrotransferred to nitrocellulose membrane and probed with specific antibodies: anti-Rad51 (Oncogene Research) and antirabbit HRP (Santa Cruz) antibodies. Antibody binding was detected with the Luminol detection kit (Santa Cruz).

### 2.7. RNA Analysis

We suspended 5 × 10^6^ cells in 1 ml of TRIzol (Invitrogen) and incubated them for five minutes. We then added 200 *μ*l of chloroform, and the extracts were centrifuged at 12000 × g for 20 minutes at 4°. The supernatant was retrieved. RNA was precipitated by adding one volume of 75% ethanol and purified with the PureLink RNA Micro kit (Invitrogen) according to the manufacturer's protocol. Reverse transcription was carried out on 500 ng of RNA, with the SuperScript III system (Invitrogen). PCR was performed with 1 *μ*l of the RT products, with the following oligonucleotides: for EP300, Forward, 5′-CTTGTTCACAAACTCGTCCAAGCC-3′ and Reverse 5′-TGTGATGGGAACTGAGTCTGAGG-3′; for ATF7IP, Forward 5′-TGCCAAAAGAAGCCTTTCTGGTCC-3′ and Reverse 5′-TCAAATACAGCACACTGCAGCGC-3′; for GAPDH, Forward 5′-ATCATCTCTGCCCCCTCTGCTGATGCCCCC-3′ and Reverse 5′-GATGACCTTGCCCACAGCCTTGGCAGCGCC-3′; for I-SceI, Forward 5′-TAATGAACCTCGGTCCGAACTCTAAACTGC-3′ and Reverse 5′-AATTTGTTACGCAGACCCTTAACCAGG-3′. PCR was stopped during the exponential phase of amplification, at 24 cycles, and the reaction mixture was loaded onto a 1% agarose gel. PCR products were quantified with ImageJ software.

## 3. Results and Discussion

### 3.1. Development of a High-Throughput Screening Assay

We developed a cell-based functional assay amenable to high-throughput screening for genome-wide screening for factors affecting nuclease-induced HGT. We constructed a reporter system based on an inactivated firefly luciferase gene, with HGT targeting this gene restoring readily detectable luciferase activity (Figure 1(a)). As luciferase inactivation resulted from the replacement of the first 22 bp of the gene with an I-SceI target site, HGT could be induced by expression of the meganuclease. This reporter construct was stably introduced into the genome of the human GM00847 cell line, and a clone carrying a single-copy insertion was identified by Southern blot analysis (data not shown). Under our experimental conditions (see Section 2), we detected no activation of the luciferase gene in cells transfected with the repair matrix alone, indicating that classical HGT was not detectable in these conditions. By contrast, transfection with a plasmid containing the repair matrix and an I-SceI-expressing cassette increased luciferase activity by a factor of 30 (Figure 1(b)).

For further validation of our assay, we cotransfected cells with the plasmid containing the repair matrix and the meganuclease-encoding cassette, together with an siRNA targeting the human *RAD51* gene (Figure 1(b)). As a negative control, we used an siRNA with no known human targets (siRNA All STAR (AS) from QIAGEN). The *RAD51* siRNA effectively knocked down the expression of its cognate target gene, as shown in Figure 1(c). As expected, it strongly inhibited (by a factor of six) I-SceI-induced HGT. The knockdown of NHEJ genes has been shown to stimulate DSB-induced recombination between chromosomal repeats [47, 51, 54]. By contrast, conflicting data have been obtained regarding the impact of NHEJ genes on classical HGT [48, 49]. We thus investigated the impact of an siRNA targeting the *LIG4* gene. In our preliminary experiment, consistent with a recent RNAi study [54], the *LIG4* siRNA was shown to increase HGT by a factor of two (Figure 1(b)). Altogether these results confirm the relevance of our experimental system for use in the search for factors affecting HGT.

### 3.2. Screening of a Genome-Wide Collection of siRNAs

**Figure 2:**
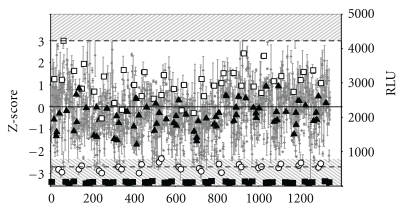
Representation of a typical primary screening run. Fourteen 96-well plates containing siRNAs for the screen and control siRNAs were transfected with RMLuc+I-SceI or pUC in duplicate. Seventy-two hours after transfection, luciferase activity was detected. Each dot represents the mean value per siRNA. The black box shows the luciferase value obtained following transfection with the empty vector (pUC), corresponding to background. Black triangles represent the values obtained for cotransfection with the AS siRNA and RMLuc+I-SceI; white circles represent the values obtained for cotransfection with *RAD51* siRNA and RMLuc+I-SceI. Finally, white squares represent the values obtained for cotransfection with *LIG4* siRNA and RMLuc+I-SceI. The black line indicates the mean value for the run. Dashed boxes show the cutoff values for hit definition.

We used our assay to screen a collection of siRNAs targeting 19,121 human genes (Qiagen). This collection included two individual siRNAs per gene, to overcome the problems of high false-positive and false-negative hit rates associated with siRNA pools and to improve confidence that the observed hits were due to silencing of the intended genes. The quality of the runs was monitored by introducing siRNAs targeting the human *RAD51* and *LIG4* genes into each 96-well plate as positive controls. A typical experiment is shown in Figure 2. The luciferase signal increased from 65 ± 15 RLU, in cells transfected with an empty vector (pUC), to 2000 ± 400 RLU following cotransfection with the plasmid containing the repair matrix and an I-SceI-expressing cassette and with the AS control siRNA. In this experiment, transient deficiencies of *LIG4* and *RAD51* genes resulted in luciferase signals of 3000 ± 450 and 550 ± 100 RLU, respectively. For all controls, the coefficient of variation (CV = *σ*/*m*, where *σ* is the standard deviation and *m* the mean of the measured values) was found to be below 20% (19%, 15%, and 19% for AS, *LIG4*, and *RAD51,* resp.) demonstrating the quality of the run. However, this level of variation is sufficient to have a significant effect on the scoring of an siRNA as positive or negative. Therefore, we decided to use a stringent cutoff point, to prevent the selection of too many false positives: siRNAs were considered as potential candidate stimulators of HGT if their *Z*-score was higher than 3. The drawback of this strategy is the risk of a high rate of false negatives. Indeed under this condition, whereas transient *LIG4* gene knockdown consistently led to the stimulation of HGT (1.4 ± 0.3), it was scored as a positive hit in only 9 of the 35 runs required to screen the entire siRNA collection (data not shown). However, the *RAD51* siRNA, which decreased HGT by a factor of six, resulted in weak signal, clearly different from the mean value of the run. These results illustrate the limits of our current screening system, which can easily miss factors having a modest effect on HGT. Moreover, the optimal timing between mRNA knockdown and the resulting phenotype may vary as a function of the gene targeted, cell type, siRNA sequence, metabolic pathway and meganuclease expression. The cotransfection strategy of siRNA and meganuclease-encoding vector could lead to a partial extinction of the targeted protein, whereas the meganuclease is at its optimum activity. However, it has been shown that treatment with siRNA targeting Ku70 and Xrcc4 reduced corresponding protein levels by 80–90% 48 h after transfection, with a return to normal levels by 96 h in HCT116 cell line (Bertolini, 2007 no. 7). Therefore, it is reasonable to think that cotransfection strategy is applicable, but it may underestimate the significance of the genes. Thus, although our primary screening assay was conducted with siRNAs targeting most of the relevant human genes, it should actually be considered far from exhaustive. 

Nevertheless, we eventually identified 290 candidates, targeting 279 different genes, from 38,242 (2 × 19,121) siRNAs, and these candidates were then subjected to secondary screening (see the following). Several potential inhibitors of HGT were also identified, and a series of siRNAs targeting 348 different genes gave levels of inhibition at least similar to the *RAD51* siRNA (signal lower than the mean signal with *RAD51* siRNA plus half its standard deviation). However, despite their important potential applications in medical fields such as cancer treatment, siRNAs involved in the inhibition of HGT were not the focus of this study and were not processed by secondary screening.

### 3.3. Secondary Screening

**Figure 3:**
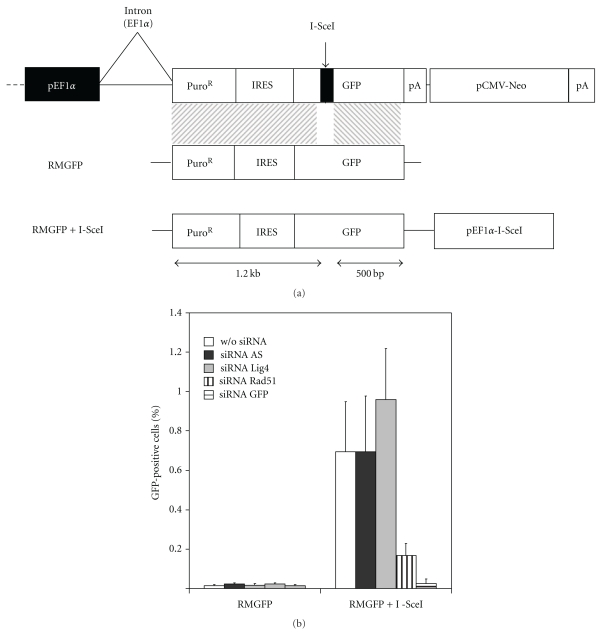
Cell-based assay for secondary screening. (a) Reporter system. The transgene used to assess I-SceI-induced gene targeting is integrated into the cGPS HEK-293 locus by expression of the I-CreI meganuclease. This transgene contains 1860 bp of the EF1*α* human promoter sequence, followed by (i) a puromycin cassette (used for selection), (ii) an IRES sequence, (iii) a *GFP* gene inactivated by the insertion of an I-SceI site generating a stop codon, (iv) a neomycin resistance sequence (used for selection) under the control of a CMV promoter. Repair plasmid and I-SceI induction (RMGFP+I-SceI) and repair plasmid alone (RMGFP) used for the induction of gene targeting by I-SceI are shown in the following, with the length of homologous sequence indicated. *GFP* reporter gene expression is assessed by flow cytometry for the quantification of gene targeting efficiency. (b) A clone resistant to both puromycin and neomycin was cotransfected with RMGFP+I-SceI or RMGFP, with or without siRNA, or with the following siRNAs: AS, *LIG4*, *RAD51*, and *GFP*. *GFP* was detected by flow cytometry 96 hours after transfection. The results of four independent experiments are presented.

High-throughput siRNA screening can lead to high false-positive rates. This problem was addressed in our experimental procedure by systematic duplication of the assay and the choice of a stringent cutoff point. However, we nevertheless designed an additional assay for secondary screening. We used the cGPS HEK-29 Full Kit (Cellectis, France) to create a cell line derived from HEK293 and carrying a single copy of the GFP coding sequence inactivated by the introduction of an I-SceI site (Figure 3(a)). Like the GM00847 derivative used in primary screening, this cell line can be used to monitor I-SceI-induced HGT. By contrast to the primary screening, this secondary screening method did not measure the global activity of a reporter gene (luciferase) in the cell population. Instead, we determined the number of cells that had acquired a functional GFP reporter gene using flow cytometry detection. Thus, siRNAs resulting in the mere enhancement of reporter gene expression should be counterselected at this step. Moreover, the two strains differ in terms of parental cell type, reporter gene, and integration locus. In this new cell line, HGT at the GFP locus was not detectable in the absence of I-SceI but was strongly stimulated when a meganuclease-encoding cassette was introduced with the repair matrix (Figure 3(b)). Consistent with our previous observations, transient *RAD51* deficiencies inhibited gene targeting efficiency by factors of 4, while LIG4 siRNA had only a mild effect, stimulating HGT by a factor of 1.4 showing that this siRNA did not lead to a strong and robust stimulation of I-SceI-induced HGT frequency.

**Figure 4:**
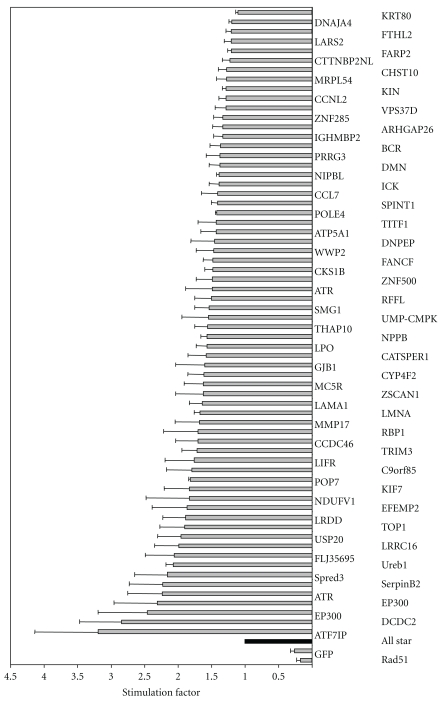
Hits selected after secondary screening. Cells were cotransfected with 200 ng of RMGFP+I-SceI and a panel of different siRNAs, as indicated, at a final concentration of 33 nM. GFP fluorescence was detected by flow cytometry 96 h after transfection. The results are expressed as the stimulation factor, corresponding to the ratio of the percentage of GFP-positive cells in the presence of the siRNA hit to that following transfection with the control siRNA (AS). Four independent experiments were performed and Student's *t*-tests were used to assess the significance of differences in stimulation factor (*P* value <  .05). Two siRNA controls were used to validate siRNA transfection: *RAD51* and GFP siRNAs. The *RAD51* and *GFP* siRNAs decreased the percentage of GFP-positive cells by factors of 6 and 4 with respect to the AS control siRNA, demonstrating that the siRNAs were active.

The 290 candidate siRNAs previously identified were tested in this new system. Sixty-six were confirmed as having a significant (*P* < .05) stimulatory effect, increasing HGT by factors of 1.2 to 3 over the siRNA control (AS). These 66 candidates corresponded to 64 different candidate genes, with the *ATR* and *EP300* genes each targeted by two different siRNAs (Figure 4). For eight genes (*ATF7IP, DCDC2, EP300, ATR, SERPINB2, SPRED3, UREB1*, and *FLJ35695*), HGT rates were more than doubled. None of the genes identified in our study was found in an esiRNA screening that was monitoring DSB-induced intrachromosomal homologous recombination [54]. Many factors may account for such discrepancies: cell type, the sequence and strength of the siRNA, and the nature of the events involved. Indeed, in our model, accessibility of the exogenous DNA repair template to the double-strand break site may be a major factor that was not addressed in this previous study since the repair template is present on the same chromatin. Therefore, our screening might identify gene involved not directly in HR mechanism but rather in regulation of HR such as the accessibility of the meganuclease and/or the exogenous DNA template to the cleavage site.

**Figure 5:**
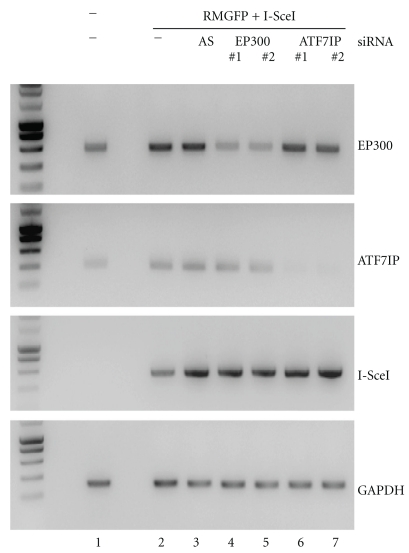
Characterization of *EP300* and *ATF7IP* knockdown. Total RNA was extracted 48 hours after transfection with the indicated DNAs and siRNAs (two different siRNA sequences were used to target each of the *ATF7IP* and *EP300* genes). RT-PCR was carried out to amplify mRNA transcribed from the *ATF7IP*, *EP300*, *GAPDH*, and *I-SceI* genes. The effects of siRNAs on their targets were monitored by analyzing band intensity after electrophoresis in 1% agarose gels. Lane 1, untransfected cells. In lanes 2 to 7, the cells were transfected with the plasmid carrying the repair matrix and the I-SceI expression cassette. Lane 2, no siRNA; lane 3, AS siRNA; lanes 4 and 5, siRNA against EP300; lanes 6 and 7, siRNA against ATF7IP.

To confirm our data and rule out possible off-target effects, new siRNAs (Invitrogen) targeting different sequences among the 21 best candidate genes were tested. As shown in Table 1, 12 of the new siRNAs significantly (*P* < .05) increased HGT, the strongest stimulation being achieved with the *ATF7IP* (4.2 ± 0.9) and *EP300* (4.2 ± 1.7) siRNAs. We further characterized these two genes. Interestingly, they have been described as transcription factors and are involved in chromatin remodeling [56–59]. RT-PCR analysis showed that the expression of these genes was knocked down by both siRNAs (Figure 5). *ATF7IP* and *EP300* mRNA levels were strongly decreased or undetectable in the presence of their cognate inhibitors, whereas no effect on GAPDH gene expression was observed. Importantly, these siRNAs had no effect on meganuclease expression, demonstrating that downregulation of the ATF7IP and EP300 genes had no impact on I-SceI expression.

Altogether, the stimulating activities on HGT, obtained for siRNAs with two cellular models and with sequences of different origins demonstrate the potential involvement of the corresponding genes on DSB-induced HGT regulation.

### 3.4. Knockdown of ATF7IP and EP300 Can Stimulate Meganuclease-Induced Gene Targeting in Endogenous Human Loci

**Figure 6:**
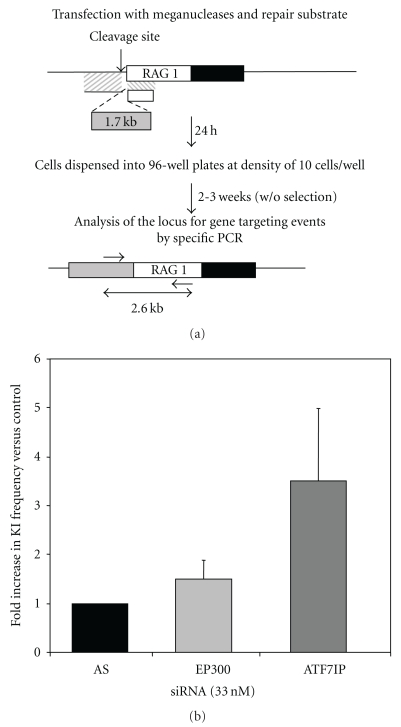
Impact of *EP300* and *ATF7IP* knockdown on the frequency of targeted integration at the endogenous *RAG1* locus. (a) Experimental design. (b) *Results*. Mean stimulation of targeted integration events. The stimulation factor is expressed with respect to control siRNA (AS). Data were obtained from 4 and 3 independent experiments with *EP300* siRNA and *ATF7IP* siRNA, respectively.

For validation of the ability of the *ATF7IP* and *EP300* siRNAs to increase HGT frequency at an endogenous locus in human cells, we used a meganuclease cleaving the human *RAG1* locus described in a previous study [55]. The principle of the targeting experiment is described in Figure 6(a). Using triple transfection with the donor repair plasmid, the meganuclease-encoding vector, and siRNA, we determined the frequency of targeted homologous recombination events with a PCR screen, as previously described [55]. This PCR screen has been validated by the characterization, by Southern blotting, of positive clones from independent experiments, with no false positive identified among more than 50 clones (data not shown). Under the experimental conditions described here, gene targeting events at the *RAG1* locus were detected in 0.7% of the transfected cells in the presence of the control siRNA (AS). By contrast, the introduction into the cells of the siRNA targeting the *EP300* gene resulted in an increase by a factor of 1.5, whereas siRNA directed against the *ATF7IP* gene increased the frequency of gene targeting events by a factor of 3.5 (Figure 6(b)).

**Figure 7:**
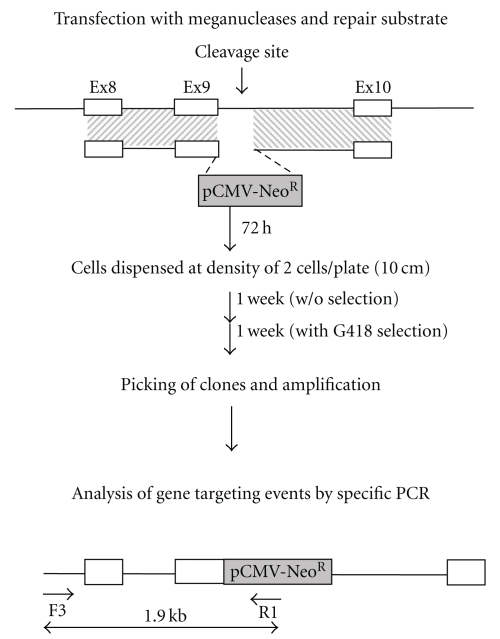
Targeted integration at the endogenous XPC4 locus: experimental design. The XPC4 target sequence is located in exon 9 of the XPC gene. Exons 8, 9, and 10 are shown in white boxes. Cleavage of the native XPC gene by the meganuclease yields a substrate for homologous recombination, which may use the repair plasmid depicted. Targeted integration events were detected by genomic PCR amplification carried out on neomycin-resistant clones.

Given the strong effect of the *ATF7IP* siRNA, we also assessed its impact on HGT at the human *XPC* locus. In this case, we used the XPC4 meganuclease, which is an optimized version of the XPC.c meganuclease described in a previous study [60]. As the frequency of DSB-induced recombination was lower at this locus (data not shown), we did not measure the absolute frequency of HGT. Instead, we measured the frequency of HGT among stable transformants: the rate of HGT versus RI of the repair matrix. This rate is used in many studies in which the absolute frequency of HGT is low, in plants [61] or mammalian stem cells [40, 62], for example. The experimental scheme is shown in Figure 7. We used a neomycin resistance (Neo^R^) cassette to select for transformants. G418-resistant clones were picked two weeks after transfection, amplified and characterized at the molecular level. Two independent experiments were conducted (Table 2). In the first, we obtained 5 targeted clones (4.2%) from 120 transformants in the presence of the AS siRNA and 15 clones (10.3%) from 146 transformants with the knockdown of *ATF7IP* expression. A second experiment performed in duplicate gave similar results, with 2 or 1 (2.4 or 1.3%) targeted clones from 83 or 73 transformants with AS, respectively, and 17 or 6 (14.9 or 10.3%) targeted clones from 114 or 58 transformants with the *ATF7IP* siRNA, respectively. These results indicate that siRNAs shown to increase I-SceI-induced gene targeting efficiency also increase the efficiency of homologous gene insertion induced by engineered I-CreI meganuclease at a natural locus.

Taken together, these data show that *ATF7IP* and *EP300,* two genes involved in transcription regulation and chromatin remodeling [56–59], can be considered as validated targets, at least for immortalized cells. Indeed, transient inactivation of these two genes may stimulate HGT at up to four different loci in human cells: the two endogenous loci, h*RAG1* and *hXPC4*, and the two endogenous luciferase and GFP reporter loci (from the primary and secondary screen). Moreover, HGT was stimulated in three different cell types (GM00847, a HEK293 derivative, and 293H). Knockdown of *ATF7IP*, the strongest candidate, gave a 3.5 times increase in HGT at the human *RAG1* locus and an up to 7 times increase at the *XPC* locus. EP300 is a histone acetylase that acetylates all four core histones in nucleosomes [63], thereby generating an epigenetic tag for transcriptional activation. ATF7IP is involved in histone methylation, another type of transcription-related epigenetic modification [56, 64]: it is required to stimulate the histone methyltransferase activity of SETDB1 [57]. SETDB1 was not among our primary hits, but it gave a 1.7 times increase in *Z*-score value in our primary screening (data not shown), indicating a stimulatory effect on the I-SceI-induced HGT luciferase signal. Altogether, these results suggest that chromatin remodelling may be an important mechanism regulating DSB-induced gene targeting. The exact mechanism of action of *ATF7IP* and *EP300* knockdown in HGT regulation remains to be determined.

## 4. Conclusion

In this study we developed a two-step screening allowing the detection of factors modulating the efficiency of I-SceI-induced HGT. We screened an siRNA collection targeting 19,000 genes. This led us to the identification of 64 genes which down regulation stimulate DSB-induced HGT frequency. For twelve of the genes we could rule out an off-target effect since an siRNA targeting the same gene but with a different sequence could still stimulate HGT frequency. Surprisingly, the gene knockdowns having the strongest impact on the efficiency of DSB-induced HGT, *ATF7IP, EP300*, are not involved in DNA-DSB repair but rather are implicated in chromatin remodelling. These data suggest an important role of these mechanisms in regulating DSB-induced gene targeting. Further characterizations of these genes would be very valuable to determine if their downregulation could still stimulate gene targeting not induced by a DSB as well as any other HR mechanisms (such as intra- or interchromosomal HR) induced or not by a double-strand break.

In addition, several other candidates not involved in chromatin remodeling, such as *ATR, LRDD, SERPINB2, UREB1*, and *TOP1,* were selected after secondary screening. It will therefore be important to assess the effect of these siRNAs on meganuclease-induced HGT at other loci. Thus, it seems likely that several other enhancers of HGT could be identified by further experiments. In principle, factors stimulating HGT by different mechanisms should have synergistic effects, making it possible to achieve much higher levels of stimulation.

## Figures and Tables

**Table 1:** Validation of the candidate genes. Candidate genes were selected after secondary screening. siRNAs targeting various mRNA sequences were tested for their ability to stimulate homologous gene targeting. Results are the means of at least 3 independent experiments.

siRNA target	Mean stimulation factor	Std	*P* value
*ATF7IP*	3.6	1.0	<.05
*EP300*	3.0	0.9	<.05
*SERPINB2*	2.5	1.1	<.05
*NDUFV1*	2.2	0.6	<.05
*LRDD*	2.0	0.6	<.05
*ATR*	2.0	0.7	<.05
*POP7*	1.8	0.6	<.05
*FLJ35695*	1.8	0.2	<.05
*DCDC2*	1.6	0.6	<.05
*RBP1*	1.5	0.2	<.05
*LIFR*	1.5	0.6	<.05
*EFEMP2*	1.4	0.2	<.05
*USP20*	1.4	0.73	>.05
*TRIM3*	1.4	0.67	>.05
*TOP1*	1.3	0.5	>.05
*HUWE1*	1.3	0.3	>.05
*SPRED3*	1.3	0.6	>.05
*C9orf85*	1.2	0.8	>.05
*LRRC16*	1.1	0.58	>.05
CCDC46	1.0	0.3	>.05
KIF7	0.9	0.4	>.05

**Table 2:** Effect on gene targeting at the *XPC4* locus of an siRNA targeting the *ATF7IP* gene.

	siRNA	Number of G418^R^ clones	Number of PCR^+^ clones	HGT (%)	Fold stimulation
Experiment 1	AS	120	5	4.2	
	*ATF7IP*	146	15	10.3	2.4
Experiment 2	AS	83	2	2.4	
	*ATF7IP*	114	17	14.9	6.2
Experiment 3	AS	73	1	1.3	
	*ATF7IP*	58	6	10.3	7.9
